# An International Validation of a Clinical Tool to Assess Carers’ Quality of Life in Huntington’s Disease

**DOI:** 10.3389/fpsyg.2019.01658

**Published:** 2019-07-23

**Authors:** Aimee Aubeeluck, Edward J. N. Stupple, Malcolm B. Schofield, Alis C. Hughes, Lucienne van der Meer, Bernhard Landwehrmeyer, Aileen K. Ho

**Affiliations:** ^1^Department of Health Psychology, School of Health Sciences, University of Nottingham, Nottingham, United Kingdom; ^2^Department of Psychology, Human Sciences Research Centre, University of Derby, Derby, United Kingdom; ^3^Human Sciences Research Centre, University of Derby, Derby, United Kingdom; ^4^European Huntington’s Disease Network, Quality of Life Working Group, Cardiff, United Kingdom; ^5^Department of Clinical Genetics, Leiden University Medical Centre, Leiden, Netherlands; ^6^Department of Neurology, Ulm University Medical Center, Ulm, Germany; ^7^School of Psychology and Clinical Language Sciences, University of Reading, Reading, United Kingdom; ^8^Department of Clinical Psychologist, Royal Berkshire NHS Foundation Trust, Reading, United Kingdom

**Keywords:** family caregiving, carers, Huntington’s disease, questionnaire, quality of life, psychometrics

## Abstract

Family carers of individuals living with Huntington’s disease (HD) manage a distinct and unique series of difficulties arising from the complex nature of HD. This paper presents the validation of the definitive measure of quality of life (QoL) for this group. The Huntington’s Disease Quality of Life Battery for Carers (HDQoL-C) was expanded (*n* = 47) and then administered to an international sample of 1716 partners and family carers from 13 countries. In terms of the psychometric properties of the tool, exploratory analysis of half of the sample demonstrated good internal consistency and reliability. Some items on the full version did not meet psychometric thresholds and a short version (HDQoL-Cs) (*n* = 23) was developed based on more stringent criteria. This was achieved using standard psychometric item reduction techniques to both increase reliability and reduce the burden of carers completing the scale. Confirmatory factor analysis of the model structure showed a good fit for all factors and indicated that the HDQoL-C and HDQoL-Cs are psychometrically robust measures of QoL. We found that carers who lived with and looked after their spouse/partner had reduced sense of coping, hope for the future, and overall QoL. Carers with children who were at risk carried the gene or were symptomatic also had poorer QoL outcomes. Findings indicated the HDQoL-C and HDQoL-Cs are valid in multiple languages and across varied cultures as measures of self-reported QoL in family carers of individual’s living with HD. These psychometrically validated tools can aid and guide the implementation of therapeutic interventions to improve life quality in this population and research into international and cross-cultural carer experiences. The HDQoL-Cs is recommended as the definitive international measure of HD carer QoL.

## Introduction

Huntington’s disease (HD) is a chronic and degenerative disorder causing movement abnormalities, cognitive deterioration, and affective disturbances (Bates et al., 2002; Quarrell, 2008; Ho et al., 2011). It is a genetic condition inherited as an autosomal dominant trait (Hartelius et al., 2010). There is currently no cure for HD, with treatments being symptomatic, palliative, or experimental in nature (Imarisio et al., 2008). It is often the immediate family that assume the responsibility of care for the individual living with HD (Aubeeluck et al., 2012). The hereditary nature of HD appears to place additional burden on patients (Ho et al., 2019) placing additional strain and a duty of care onto carers (Williams et al., 2000) impacting on their quality of life (QoL) (Aubeeluck et al., 2012). In HD the carer journey is one that is decades long, with implications for the partner or eventual carer early on in the piece, given the impact of genetic testing for HD and also behavioral and cognitive changes which may even precede overt motor symptomatology and diagnosis. Partners will often have the added burden of knowing the risk for their offspring. Caring for an individual living with HD and raising children who have a 50% risk of eventually developing HD is emotionally challenging and may lead to increased worry compared to other, non-genetic diseases (Ho et al., 2019). In particular, HD carers report burden, isolation, financial pressures (Semple, 1995), difficulty in coping (Carreon et al., 2018), the management of cognitive/behavioral symptoms (Simpson et al., 2016), and emotional distress (Williams et al., 2009) as associated with their caregiving role.

There has been a steady growth in work that explores the impact of HD on the QoL of family carers. The existing literature would argue that health and life quality are reduced in this carer group (e.g., Kessler, 1993; Ready et al., 2008; Williams et al., 2009; Hartelius et al., 2010; Skirton et al., 2010; van Walsem et al., 2017). Mestre et al. (2018) argue that health-related quality of life (HRQoL) is an important outcome measure that can form part of the core clinical assessment of individual’s living with HD and their carers. They rated the Huntington’s Disease Quality of Life Battery for carers (HDQoL-C; Aubeeluck and Buchanan, 2005, 2007) as a “suggested” tool for use with family carers of such individuals but not as a “recommended” measure and called for further validation to be completed across the measures they considered. The present paper addresses this issue.

Aubeeluck et al. (2013) further explored the properties of the HDQoL-C in French and Italian translations. This study found additional evidence for the reliability and validity of the scale in a cross-cultural sample and also showed a differing factor structure from the Aubeeluck and Buchanan (2007) study. Thus indicating the need for further international validation of QoL measures for HD carers to devise a generalizable cross-cultural measure.

The Enroll-HD clinical research database presented an opportunity to revalidate the HDQoL-C (Aubeeluck and Buchanan, 2007) with an international sample of partners and family carers to allow for the development of a truly international clinical tool for the measurement of QoL in HD carers. As this expanded measure is widely used but has not previously been subjected to psychometric scrutiny it was important to examine the reliability of the expanded HDQoL-C. To this end, the present study assessed the psychometric components of an expanded version of the HDQoL-C (developed in collaboration with the European HD QoL Working Group) and explored self-reported QoL in family carers of individuals living with HD to support the goals of screening and severity testing in the HD carers population using principal axis factoring (PAF). The psychometric properties of the revalidated HDQoL-C were then scrutinized with the goal of refining the measure based on psychometric item reduction procedures. The paper then examines the case for the resulting short-form of the scale which would have obvious benefits for carers if equivalent or increased reliability were to be demonstrated. The factor structures were finally tested with confirmatory factor analysis (CFA). Demographic and situational effects on QoL were also explored to test the validity of the tool. Thus, the aims of this study were to (a) evaluate the psychometric properties of the expanded version of the HDQoL-C and (b) identify whether a more psychometrically robust, shorter version of the scale could be derived for future use in clinical practice and research.

## Materials and Methods

### Participants

Enroll-HD is a global clinical research platform designed to facilitate clinical research in HD. Core data sets are collected annually on all research participants as part of this multi-center longitudinal observational study of HD. Carers who participate in Enroll-HD complete the HDQoL-C during annual study visits as part of their participation. This project is ongoing but at the time of data retrieval, a total of 1716 partners/carers were participating in this multi-center international study. The mean age of participants was 52.8 (*SD* = 13.1), 59.5% of the sample were female and 40.4% were male, 0.1% did not respond to the question. They were recruited as part of the Enroll-HD observational study of HD, where partners/carers also provide information. The Enroll-HD study collected data from Argentina, Australia, Canada, Denmark, France, Germany, Italy, New Zealand, Netherlands, Poland, Spain, United Kingdom, and the United States.

Data are monitored for quality and accuracy using a risk-based monitoring approach. All sites are required to obtain and maintain local Ethics Committee approvals. Enroll-HD recruits through specialty clinics involved in HD who have ethical approval for this study1^1^. Oversight of the local approvals is from the Enroll ethics working group^2^ who ensure that the Enroll project achieves full compliance with national and regional Ethics, Scientific Appraisal for data release, and Data Transfer regulation legislation and best practice. The data included in the manuscript were obtained as part of the Enroll project, and Enroll-HD have approved the data to be used in this paper (reference code SPS017). All participants provided written informed consent to take part and are free to withdraw at any point without any implications on care.

### Questionnaire

The HDQoL-C was developed using data gathered from three preliminary qualitative studies (Aubeeluck, 2005; Aubeeluck and Buchanan, 2006). Study 1 gathered carer ratings on the existing domains and facets of the COMQOL-A5 (Cummins, 1997), a well-documented and validated tool for measuring QoL with the general adult population. Study 2 utilized photovoice methodology to capture carers experiences of living with HD. Study 3 investigated the emerging themes from studies 1 and 2 in a focus group setting drawing on both carer and professional perspectives. The HDQoL-C examines the caregiving experience of family carers and/or partners of patients living with HD. It is a multidimensional, disease-specific, and subjective HR-QoL tool that incorporates the individual’s physical health, psychological state, level of independence, social relationships, and personal beliefs. The original pilot version of the HDQoL-C contained 63 items for exploration that were reduced to 34 via psychometric analysis. Participants respond to statements on a 10-point Likert scale to indicate the extent to which they agree with the statement. Cronbach’s alpha scores for the three components of the original HDQoL-C scale demonstrate good internal consistency – 0.801 (practical aspects of caregiving), 0.844 (satisfaction with life), and 0.885 (feelings about living with HD), with test re–test reliability for the same components being 0.86, 0.90, and 0.92, respectively (Aubeeluck and Buchanan, 2007).

In this current study, all 63 original items were put forward to the European HDQoL Working Group and these were discussed for relevance to a European population and further additions to the scale were made. The European HDQoL Working Group carer questionnaire, a 47-item modified version of the HDQoL-C was distributed for use in the Enroll clinical database.

The English questionnaire originating from the United Kingdom was translated into all other languages using an EN 15038-certified translation process involving dual forward translation including reconciliation and back translation. Translations were made by members of the QoL working group with a background in HD care who were proficient users of English (Common European Framework of Reference – Level C1 or C2) and native speakers in the goal language. Different United Kingdom-English, US-English, Australian-English, Canadian-English versions, and Spanish–Spanish and US–Spanish versions were translated to reflect specific regional and cultural differences within a particular language where appropriate.

### Analytic Strategy

For the purposes of the analysis, the sample of 1716 partners/carers was randomly allocated to two equal groups (*N*s = 858): the first for the exploratory analyses and the second for the confirmatory analyses.

Due to there being minimal psychometric testing on the expanded HDQoL-C a standard psychometric process was applied to the data. First, items were screened for normality and item-total correlations to determine the extent to which individual items correlated with the total score on the scale were completed for both sections of the scale. Exploratory factor analyses (EFAs) were conducted for each section to identify subscales measuring different aspects of QoL. The resulting sub-scales were then tested for reliability using Cronbach’s alpha.

The items on from the expanded HDQoL-C scale were treated as the item pool from which to develop a refined shorter version of the scale for ease of use in future clinical practice and research. The psychometric process outlined above was repeated to develop the short form (HDQoL-Cs) of the tool except more stringent criteria were applied to remove items that did not correlate with the total, or violated assumptions of normality. Nunnally and Bernstein’s (1994) criteria for item-total correlations were applied to remove items. Comrey and Lee’s (1992) minimum threshold for factor loadings of 0.45 and maximum factor cross-loadings of 0.3 were set and iterative EFAs were completed to remove items that did not meet these standard psychometric threshold criteria.

The final factor structures of the HDQoL-C and HDQoL-Cs were then tested in confirmatory analysis to determine whether these structures were a good fit for these data.

## Results

### Exploratory Factor Analysis

In order to test the efficacy of the scale as currently used to assess HD carers’ QoL in clinics across the world all items were retained in the first EFA. Item total correlations were calculated for both sections.

#### Section 1

In Section 1, no items had an item-total correlation of *r* < 0.3 so all items met the criteria set by Nunnally and Bernstein (1994). However, Section 2 had three items that did not meet this criteria and could be candidates for exclusion: “I feel that Huntington’s Disease brought something positive to my life” (*r* = 0.186), “I feel that my own needs are important to others” (*r* = 0.276), and “I feel that Huntington’s Disease has made me a stronger person” (*r* = 0.178). These items were retained in the present analysis to explore and confirm the factor structure in the currently utilized clinical tool.

Exploration of univariate descriptive statistics for candidate items revealed evidence of outliers and skew and kurtosis for some variables; therefore, PAF with a direct oblimin rotation were used for factor extraction. Separate PAF analyses were completed to explore each section of the scale – Section 1 was concerned with carer’s life satisfaction and Section 2 was concerned with carer’s feelings about themselves and others.

The Kaiser–Meyer–Olkin (KMO) measure of sampling adequacy showed the data for Section 1 to be factorable (KMO = 0.856), Bartlett’s test of sphericity was also highly significant (χ^2^ = 2868, *df* = 36, *p* < 0.001). Low off-diagonal values in the anti-image correlation matrix also indicated that the data were suitable for factor analysis (Tabachnick and Fidell, 2007). Eigen values and the Scree plot both indicated a two factor solution with six items loading on to Factor 1 and three items loading on to Factor 2. No problematic cross-loadings were identified (cross-loadings >0.2 are reported in the pattern matrix in Table 1).

**TABLE 1:** Pattern matrix for Section 1 – satisfaction with life.

**Retained items**	**Factor 1**	**Factor 2**
How satisfied are you with feeling a part of your social environment?	**0.879**	–
How satisfied are you with your relationships with your friends?	**0.742**	–
How satisfied are you with your psychological health?	**0.687**	–
How satisfied are you with what you have achieved in life?	**0.646**	–
How satisfied are you with family relationships?	**0.586**	–
How satisfied are you with your physical health?	**0.570**	–
How satisfied are you with the professional support you receive?	–	**0.815**
How satisfied are you with the medical treatment that your HD relative(s) receive(s)?	–	**0.811**
How satisfied are you with the way other people behave toward the HD person?	0.264	**0.344**

*Bold font denotes the factors that items load on to most strongly.*

#### Section 2

For Section 2, the KMO measure of sampling adequacy showed the data to be factorable (KMO = 0.926), Bartlett’s test of sphericity was also highly significant (χ^2^ = 14,339, *df* = 703, *p* < 0.001). Low off-diagonal values in the anti-image correlation matrix also indicated that the data were suitable for factor analysis (Tabachnick and Fidell, 2007). Initial analysis suggested eight factors with Eigen values greater than one. The pattern matrix showed many items with cross loadings and Factor loadings below the minimum 0.32 specified by Tabachnick and Fidell (2007) suggesting a problematic factor structure. Costello and Osborne (2005) described the Eigen values >1 criteria as a highly inaccurate rule of thumb. We therefore used the Scree plot to identify three factors, the PAF analysis was then re-run with this number of factors which produced a more parsimonious factor structure. The pattern matrix for this analysis is shown in Table 2. There were 14 items in Factor 1, 13 in Factor 2, and 10 in Factor 3. Some items showed cross-loadings >0.3 and some factor loadings did not meet the minimum criteria for inclusion. Nonetheless, reliability analyses showed that the factors were internally consistent: all Cronbach’s alphas were >0.8.

**TABLE 2:** Pattern matrix for Section 2 – feelings about living with HD.

**Retained items**	**Factor 1**	**Factor 2**	**Factor 3**
I feel sad	**0.848**	–	–
I feel depressed	**0.805**	–	–
I feel a sense of grieving	**0.754**	–	–
I feel a sense of loss	**0.715**	–	–
I feel stressed	**0.694**	–	–
I feel exhausted	**0.682**	–	–
I feel a sense of anguish	**0.673**	–	–
I feel full of fear	**0.573**	–	–
I feel lonely	**0.459**	0.218	0.211
I feel guilty	**0.445**	–	
I feel isolated	**0.425**	–	0.235
I feel financially disadvantaged	**0.385**	–	–
I feel I get enough sleep	**0.348**	0.254	–
I feel worried about the genetic consequences of HD	**0.265**	–	–
I feel that HD has made me a stronger person		**0.625**	
I feel supported	–	**0.619**	–
I feel that my role as a carer is rewarding	–	**0.615**	–
I feel satisfied with my overall QoL	0.332	**0.577**	–
I feel that I can cope	0.207	**0.577**	–
I feel there is hope for the future	0.230	**0.547**	–
I feel comforted by my beliefs (religious, philosophical, or spiritual)	–	**0.518**	–
I feel I have somebody to turn to for assistance if I am overwhelmed	–	**0.484**	–
I feel happy	0.397	**0.484**	–
I feel that HD brought something positive to my life	–	**0.459**	–
I feel safe	0.265	**0.449**	–
I feel that my own needs are important to others		**0.344**	–
I feel I have enough time for myself	0.237	**0.332**	–
I feel embarrassed by the behavior of my HD relative(s)	–	–	**0.819**
I feel ashamed of the behavior of my HD relative(s)	–	–	**0.781**
I feel restricted by having to provide continuous care	–	–	**0.497**
I feel restricted by the need to maintain secrecy about HD in the family	–	–	**0.493**
I feel resentful	0.228	–	**0.489**
I feel threatened		–	**0.454**
I feel restricted by a regimented daily routine	0.226	–	**0.429**
I feel that I have had a duty of care forced on me		–	**0.404**
I feel frustrated by the discrimination of others toward my HD relative(s)	0.219	–0.215	**0.395**
I feel frustrated by the misconceptions of others toward my HD relative(s)	0.275	–0.218	**0.378**
I feel like I don’t know who I am anymore	0.350		**0.354**

*Bold font denotes the factors that items load on to most strongly.*

These analyses show that while the scale meets some threshold standards, there are a number of sub-optimal items included that undermine the psychometric properties of the scale.

In the next section, we describe the application of more stringent psychometric criteria and detail the item reduction procedure by which items were excluded, first through violations of normality, then item-total correlations, and finally by excluding all items that did not meet Comrey and Lee’s (1992) “fair” criteria: minimum threshold for factor loadings of 0.45 and maximum factor cross-loadings of 0.3. This approach resulted in a shortened version of the scale.

### Short Version HDQoL-Cs

The HDQoL-C is currently in use both in clinical settings and for research purposes; however, the preceding analysis demonstrated that the scale has some sub-optimal psychometric properties. The following section details the process of refining the items through a standard psychometric process to produce a shorter more reliable version of the scale.

Scrutiny of items for assumptions of normality revealed that the item “I feel threatened” was highly skewed and kurtosed as very few participants indicated that they felt threatened (67% answered with the lowest possible level of threat on the 10-point Likert scale and only 6.7% reporting a level of threat higher than the mid-point on the scale), as a result this item was removed. Item-total correlations were conducted and found three problematic items with *r* < 0.3, these items were removed and all item-total correlations then met the threshold for inclusion in the PAF analysis.

#### Section 1

For section 1, the KMO measure of sampling adequacy showed the data to be factorable (KMO = 0.838) and Bartlett’s test of sphericity was also highly significant (χ^2^ = 2653, *df* = 28, *p* < 0.001). PAF analysis revealed two factors with one item in Factor 2 (How satisfied are you with the way other people behave toward the HD person) not meeting the criteria of Factor loadings >0.45 with no cross loadings >0.3. Thus, Section 1 retained all but one item for the shortened version of the questionnaire. See Table 3 for the factor structure.

**TABLE 3:** Pattern matrix for satisfaction with life – short form.

**Retained items**	**Factor 1**	**Factor 2**
How satisfied are you with feeling a part of your social environment?	**0.866**	–
How satisfied are you with your relationships with your friends?	**0.747**	–
How satisfied are you with your psychological health?	**0.689**	–
How satisfied are you with what you have achieved in life?	**0.655**	–
How satisfied are you with family relationships?	**0.591**	–
How satisfied are you with your physical health?	**0.577**	–
How satisfied are you with the professional support you receive?	–	**0.829**
How satisfied are you with the medical treatment that your HD relative(s) receive(s)?	–	**0.766**

*Bold font denotes the factors that items load on to most strongly.*

#### Section 2

For Section 2, the KMO measure of sampling adequacy again showed the data to be factorable (KMO = 0.927) and Bartlett’s test of sphericity was also highly significant (χ^2^ = 13,427, *df* = 595, *p* < 0.001). Initial analysis suggested six factors with Eigen values >1. As with the first analysis, however, we used the Scree plot to identify a three-factor solution and the PAF analysis was re-run specifying these factors. The pattern matrix again showed items with cross-loadings >0.3 and some factor loadings did not meet the minimum criteria for inclusion. The recommended criterion value of 0.45 was then applied to the factor loadings and all items that did not meet this threshold were excluded. Items with cross-loadings of >0.3 were also removed. Fourteen items were removed in the first EFA. This approach was repeated until all remaining items in the final iteration met these criteria. The PAF analysis for the final set of items is reported below. See Table 4 for the factor structure.

**TABLE 4:** Pattern matrix for feelings about living with HD – short form.

**Retained items**	**Factor 1**	**Factor 2**	**Factor 3**
I feel sad	**0.830**	–	–
I feel depressed	**0.769**	–	–
I feel a sense of grieving	**0.741**	–	–
I feel a sense of loss	**0.722**	–	–
I feel exhausted	**0.692**	–	–
I feel stressed	**0.692**	–	–
I feel a sense of anguish	**0.662**	–	–
I feel full of fear	**0.551**	–	–
I feel that I can cope	–	**0.668**	–
I feel there is hope for the future	–	**0.614**	–
I feel satisfied with my overall QoL	0.273	**0.598**	–
I feel supported	–	**0.589**	–
I feel comforted by my beliefs (religious, philosophical, or spiritual)	–	**0.588**	–
I feel that my role as a carer is rewarding	–	**0.578**	–
I feel safe	–	**0.524**	–
^*^I feel ashamed of the behavior of my HD relative(s)	–	–	**0.894**
^*^I feel embarrassed by the behavior of my HD relative(s)	–	–	**0.861**

^*^Excluded from final version of feelings about living with HD – short form due to CFA outcomes. Bold font denotes the factors that items load on to most strongly.

The final model showed strong sampling adequacy (KMO = 0.885) and Bartlett’s test of sphericity was also highly significant (χ^2^ = 6672, *df* = 136, *p* < 0.001). Factor 1 included eight items and was indicative of negative emotions such as grieving, loss, stress, and exhaustion. Factor 2 included seven items and was more positively framed, with items focused on life satisfaction, the rewarding aspects of caring, coping, and hope for the future. Factor 3 only had two items but these were both robust (factor loadings > 0.8) and were focused on shame and embarrassment about the behavior of the HD relative. Reliability tests found Cronbach’s alpha = 0.900 for Factor 1, Cronbach’s alpha = 0.807 for Factor 2, and Cronbach’s alpha = 0.863 for Factor 3, with no candidate items for removal in any factor. The short form of the tool was named HDQoL-Cs.

### Confirmatory Factor Analysis

Missing data were handled using the data imputation method predictive mean matching (PPM), using the Mice package in R. CFA was conducted on the HDQoL-C and HDQoL-Cs. Proposed models of the factor structures shown by the PAF analysis were tested: For the HDQoL-C Model 1 tested Section 1, “Satisfaction with life” from the full version of the HDQoL-C. The factor structure that was tested had nine items and two factors. Model 2 tested Section 2, “Feelings about living with HD” from the full version of the HDQoL-C which had 38 items and 3 factors.

The factor structure for the HDQoL-Cs was also tested with Model 3 testing the shortened version of “Satisfaction with life.” Model 4 tested the shortened version of “Feelings about living with HD” which had 17 items and 3 factors. Sampling adequacy was “marvelous” (Hutcheson and Sofroniou, 1999; Field, 2013) according to the KMO measure for all models (Table 5). Bartlett’s test of sphericity was highly significant (Table 5) indicating the correlation matrix is factorable. The sample size was >200 (*N* = 858) and more than adequate for a CFA (Worthington and Whittaker, 2006). Therefore, the maximum-likelihood method was employed and the first variable in each subscale was set as one for data scaling purposes.

**TABLE 5:** Model fit for HDQoL-C and HDQoL-Cs.

**Models**	**χ^2^**	***df***	***p***	**RMSEA**	**SRMR**	**CFI**	**TLI**	**NFI**	**KMO**	**Bartlett’s test**
1. Satisfaction	119.215	22	<0.001	0.071	0.045	0.971	0.955	0.965	0.866	$\chi_{\left(36\right)}^{2}$ = 3144.50, *p* < 0.001
2. Feelings	2329.95	655	<0.001	0.056	0.060	0.873	0.864	0.832	0.926	$\chi_{\left(595\right)}^{2}$X = 11,843, *p* < 0.001
3. Satisfaction (short)	85.013	16	<0.001	0.072	0.033	0.977	0.960	0.972	0.850	$\chi_{\left(28\right)}^{2}$ = 2871.1, *p* < 0.001
4. Feelings (short)	193.069	84	<0.001	0.040	0.036	0.980	0.975	0.966	0.892	$\chi_{\left(136\right)}^{2}$ = 5913, *p* < 0.001
Cut-off values (Hu and Bentler, 1999)	N/A	N/A	>0.05	<=0.06	<=0.08	>=0.95	>=0.95	>=0.95		

There were no negative error variances (Heywood Cases) in Models 1 (“Satisfaction with life”), 2 (“Feelings about living with HD”), or 3 (“Satisfaction with life” short), and none of the squared multiple correlations (SMCs) exceeded 1. Factor loadings were examined for each model; for Model 1 the lowest loading was the item “How satisfied are you with the way other people behave toward the HD person?” (*r*^2^ = 0.33). The item reduction process for the shortened form removed the weakest item from Model 3 (“Satisfaction with life” – short) and the revised model fit is reported in Table 5. The weakest item in Model 2 was “I feel that HD has made me a stronger person” (*r*^2^ = 0.09). The EFA for Model 2 included a substantial number of problematic items that were removed in creating HDQoL-Cs, Model 4 tests the resultant factor structure.

Model 4 (“Feelings about living with HD” – short) could not be computed due to the presence of an extreme Heywood case in Factor 3. Given that this was a two-item factor (which can be considered psychometrically problematic in itself) the analysis was re-run on a two factor solution with 15 items – excluding “I feel ashamed of the behavior of my HD relative(s)” and “I feel embarrassed by the behavior of my HD relative(s)” in order to render the model computable. Thus, the fit measures in Table 5 are for a two-factor solution to the “Feelings about living with HD” section.

Initial CFAs showed all of the fit indices were beyond the recommended thresholds with values indicating that the models were a mediocre fit for the data. However, covariates were identified in each model and the confirmatory models were adjusted to include covariance between items. A number of measures of fit were examined for the re-specified models (Table 5) taken from various types of fit indices including: overall fit (Chi-square), absolute fit (SRMR and RMSEA), and incremental fit (NFI, CFI, and TLI). Chi-square fit tests were significant although the sample size makes this highly likely even when the data fit the model well. Models 1 and 2 showed a much improved fit, with most other estimates within acceptable levels. Models 3 and 4 showed a good fit with all thresholds criteria being met except for RMSEA for Model 3. The HDQoL-Cs showed improved levels of fit compared to the HDQoL-C with most fit statistics showing improvements for the shortened version over the original full-length version.

### Inferential Analyses – Validation

Inferential tests were conducted to examine differences in reported QoL for carers with differing personal circumstances using the HDQoL-Cs as an outcome variable. Due to uneven sample sizes and outliers in all analyses non-parametric tests of difference were employed throughout. In each analysis, the total score for Section 1 and Section 2 was compared between groups. To control for the number of comparisons, the threshold alpha level for significance was set to 0.01. For each validation test, we first compared groups across the overall scales (satisfaction with life and feelings about living with HD). Where differences were found for the overall scores these were followed up to examine differences on the relevant sub-scales.

Spousal carers were compared with all other categories of carer using Mann–Whitney *U*-tests and no difference was found for overall satisfaction with life *Z* = −0.493 (*N*_1_ = 520, *N*_2_ = 1123), *p* = 0.622. However, significant differences were shown for total score for feelings about living with HD *Z* = −2.729 (*N*_1_ = 509, *N*_2_ = 1093), *p* = 0.006 (spousal carers: median = 110, IQR = 42.0; other carers: median = 114, IQR = 43.0). Analyses of the subscales showed that Subscale 1 was not significant (*p* = 0.657); however, Subscale 2 showed a significant contrast *Z* = −2.729 (*N*_1_ = 519, *N*_2_ = 1109), *p* < 0.001 such that carers who looked after their spouse/partner had reduced average sense of coping, hope for the future, and overall QoL (spousal carers: median = 44, IQR = 20; other carers: median = 48, IQR = 18).

These analyses were then repeated for carers whose parent was the HD patient. Mann–Whitney *U*-tests found no difference for overall satisfaction with life *Z* = −0.499 (*N*_1_ = 175, *N*_2_ = 1468), *p* = 0.618. However, significant differences were shown for total score for feelings about living with HD *Z* = −3.087 (*N*_1_ = 176, *N*_2_ = 1426), *p* = 0.002 (carers for parents: median = 116.3, IQR = 41.0; other carers: median = 109.96, IQR = 42.0). Analyses of the subscales showed that for this group it was Subscale 1 (carers for parents: median = 53.14, IQR = 28.0; other carers: median = 48.12, IQR = 28.0) that was significantly different to other carer groups *Z* = 3.180 (*N*_1_ = 178, *N*_2_ = 1496), *p* = 0.001; however, Subscales 2 (*p* = 0.165) and 3 (*p* = 0.882) did not differ significantly. This indicated that carers of parents experienced lower scores for negative emotions and experiences of grieving, loss, and exhaustion when compared to other groups.

Carers for their children showed a different pattern to carers for parents in that significant differences were shown in total feelings score *Z* = −3.995 (*N*_1_ = 201, *N*_2_ = 1401), *p* < 0.001 (carers for children: median = 103.35, IQR = 43.0; other carers: median = 111.72, IQR = 40.0). Analyses of the subscales also showed differences for Subscale 1, *Z* = 6.260 (*N*_1_ = 206, *N*_2_ = 1468), *p* < 0.001 (carers for children: median = 41.31, IQR = 26.0; other carers: median = 49.72, IQR = 27.0); however, Subscales 2 (*p* = 0.687) and 3 (*p* = 0.695) did not differ significantly. The differences in life satisfaction did not pass the 0.01 threshold set for significance, but were the largest observed in these analyses, *Z* = 2.02 (*N*_1_ = 205, *N*_2_ = 1438), *p* = 0.036 (carers for children: median = 59.79, IQR = 20; other carers: median = 61.83, IQR = 16.0). These difference would indicate that carers who are caring for their children have more negative emotional experiences.

Carers who lived with the HD patient were compared with those who did not using Mann–Whitney *U*-tests and no difference was found for satisfaction with life *Z* = −1.572 (*N*_1_ = 346, *N*_2_ = 1294), *p* = 0.116. However, significant differences were again shown for measures of feelings about living with HD *Z* = −4.504 (*N*_1_ = 339, *N*_2_ = 1260), *p* < 0.001. (living with HD patient: median = 109, IQR = 43.0; not living with HD patient: median = 116, IQR = 39.0). Analyses of the subscales showed that all were significantly different between those who cared for and lived with the HD patient Subscale 1 (*p* = 0.006, living with HD patient: median = 49.0, IQR = 28.75; not living with HD patient: median = 54, IQR = 25.0) and Subscale 2 (*p* < 0.001, living with HD patient: median = 45, IQR = 20.0; not living with HD patient: median = 48, IQR = 18.0).

Comparisons were made between carers who had children who were at risk, carried the gene, or were symptomatic. No difference was found for satisfaction with life *Z* = −0.059 (*N*_1_ = 655, *N*_2_ = 977), *p* = 0.953. However, significant differences were again shown for measures of feelings about living with HD *Z* = −4.514 (*N*_1_ = 635, *N*_2_ = 956), *p* < 0.001 (children who are at risk/carrier/symptomatic: median = 108, IQR = 43.0; no children who are at risk/carrier/symptomatic: median = 117, IQR = 42.0). Analyses of the subscales showed that feelings Subscale 1 (*p* < 0.001) were significantly worse for carers with children who were at risk, carried the gene, or were symptomatic (children who are at risk/carrier/symptomatic: median = 47.0, IQR = 28.0; no children who are at risk/carrier/symptomatic: median = 54.0, IQR = 26.0). There was no difference for Subscale 2 (*p* = 0.098).

Weak, negative correlations were found between duration in the caring role and total score for life satisfaction (*r*_s_ = −0.066, *N* = 1563, *p* = 0.009) and feelings about living with HD (*r*_s_ = −0.072, *N* = 1602, *p* = 0.005). Two sub-scales were significantly correlated with duration in the caring role. Feelings Subscale 1 (*r*_s_ = −0.132, *N* = 1583, *p* < 0.001) and satisfaction Subscale 1 (*r*_s_ = −0.076, *N* = 1609, *p* = 0.002). All other subscales *p* > 0.2.

## Discussion

The present study allowed for the examination and refinement of a clinical tool to measure the QoL of carers of individual’s living with HD. This was conducted as part of an international study of the largest sample of HD carers/partners that has been possible to date and resulted in a psychometrically robust tool to measure the QoL experienced by these individuals. Based on the present study, the HDQoL-C and HDQoL-Cs can be considered the definitive measures of carer/partner QoL and can be utilized in any such population across the world. The validation process will go some way to address the concerns from Mestre et al. (2018) in their call for further validation of QoL measures for HD patients and their carers. Full versions of HDQoL-C and HDQoL-Cs and the scoring methods can be found in the Appendix.

The analyses showed that the HDQoL-C was satisfactory with regards to most standards in EEFs. Section 1, “Satisfaction with life” identified two factors (satisfaction with self and personal relationships and satisfaction with the behavior of health professionals and others) and was considerable more robust than Section 2, “Feelings about living with HD.” Section 2 included three factors (negative emotions such as sadness and loss, positive emotions such as hope for the future and coping and thirdly shame and embarrassment), but these included items that did not meet standard psychometric thresholds in scale development (see e.g., Comrey and Lee, 1992; Tabachnick and Fidell, 2007).

Based on these outcomes a short version of the scale was developed and tested to determine whether the psychometric properties could be improved and whether a more efficient and convenient tool could be produced. Section 1 remained largely intact, with only one item failing to meet the threshold requirements. However, Section 2 was substantially reduced in length once items that were problematic in terms of non-normality, item-total correlations, weak factor loadings, and high cross loadings were removed. This resulted in a 23-item short form of the HDQoL-C (HDQoL-Cs) which also had improved psychometric properties. The short form continued to have two factors in Section 1 and three factors in Section 2. The expanded version of the HDQoL-C differs from the 2007 and 2013 remodeling, in that although “Satisfaction with life” and “Feelings about living with HD” are retained, “Practical aspects of caregiving,” a factor established in previous versions is now situated within “Satisfaction with life.” This might suggest that while the practicalities of caregiving are important to this population (see also Rothing et al., 2015), satisfaction and feelings in a carer/partner role take precedence over such practicalities.

Confirmatory factor analysis suggested a good fit with the data, although it also indicated that the three factor solution to the shortened “Feelings about living with HD” scale was not computable and may be best presented with two subscales rather than three. There was evidence that items covaried with strong correlations between, for example items asking about grieving and loss. The was also a strong relationship observed between satisfaction with psychological health and satisfaction with friendships which emphasizes the importance of social support for good mental health outcomes among carers and warrants further research in the future.

The good fit and positive psychometric outcomes observed were in spite of the diversity of the population tested when compared with the United Kingdom population recruited in the original development of the HDQoL-C. Aubeeluck et al. (2013) showed that there were differing factor loadings from the United Kingdom sample in a French and Italian sample. The factor structure in the present study, however, differed from both the original and shortened French/Italian version and cultural differences in the populations involved may have impacted on the factor structure and model fit. As such, the expanded version of the HDQoL-C and HDQoL-Cs may not fully capture the nuanced variation in factor structure across cultures. The present data set did not offer the opportunity to make such cross-cultural or language comparisons which could be considered a limitation of the study. There is, however, clearly a benefit to having a generalized international tool that allows cross-cultural comparisons of standards of QoL between HD partners/carers in different locations. A standard international measure will facilitate these comparisons, and it remains that further studies to unpack cultural differences in experiencing life as an HD carer are required.

Analysis utilizing the short-form of the questionnaire to examine differences based on demographic and individual circumstances demonstrated that the burden of caregiving was, on average, greater for spouses and partners than for other family members. Caregiving impacted on feelings to a greater extent for those living with the HD patient. Carers who had children who either had the potential to develop HD, carried the gene or were symptomatic had lower scores for Section 2 of the scale, indicating a greater sense of loss and sadness. Such data indicate that these groups would benefit from greater emotional and psychological support and further the argument for psychological interventions as standard for partners and/or carers of individual’s living with HD.

While many of the items on the scale are not explicitly HD specific, it is important to note the implicit complexity of latent variables and the ability of these to measure their impact on the HD carers QoL. Through the qualitative exploration of what matters for HD carers in terms of their life quality, we know that the impact of caregiving in HD has a variety of unique and distinct features that set it apart from other types of family caregiving. For example, the genetic nature of HD means that it is not unusual for children to care for their parents before presenting with symptoms themselves or for parents to care for a spouse and then a child over a number of generations. Such a chronic and extended caregiving role can exacerbate the isolation, sense of loss for their family members and their own future, feelings of guilt, anger, burden, and disempowerment for HD families (Aubeeluck et al., 2012). Therefore, while for example, “genetic issues” are not explicit within the scale, the impact of these in terms of “sense of loss” is clear. These genetic issues are further evidenced by the increased negative feelings experienced by parents caring for their children.

In summary, the present study has demonstrated that the clinical tool devised to measure QoL in HD partners/carers has a good factor structure and showed merit in distinguishing different groups of HD carers in terms of their lived experience. The development of the short form of the questionnaire showed a slightly more reliable factor structure, but more importantly reduced the burden of completing the instrument by reducing the questions to be completed from 46 to 23, which is an important consideration for a group with challenging life circumstances. The measures validated here will facilitate the examination of the complex issues that impact upon HD carers (e.g., Domaradzki, 2016) and are already of use in clinical practice and research internationally. We therefore recommend the use of the HDQoL-Cs as a measure of self-reported QoL in carers of individual’s living with HD, but note that the expanded version has adequate psychometric properties for continued use. The HDQoL-Cs can help clinicians to understand and support carers in their role, facilitate carers engaging in self-reflection, and encourage the investigation of novel research questions. We therefore argue that HDQoL-Cs should be considered the definitive measure of HD carer QoL for researchers and clinicians due to its improved ease of use and psychometric properties. This international validation further supports international and cross-cultural comparisons of QoL using a common metric which has not previously been possible. These psychometrically validated tools can aid and guide the implementation of therapeutic interventions to improve life quality in this population.

## Ethics Statement

Data were collected as part of the Enroll-HD project and conformed with their requirements. Data were monitored for quality and accuracy using a risk-based monitoring approach. All sites were required to obtain and maintain local Ethics Committee approvals. Enroll-HD recruits through specialty clinics involved in HD who have ethical approval for this study. Participants provided written informed consent to take part and were free to withdraw at any point without any implications on care. IRB/IEC approval consistent with local regulations was obtained for each site. Prior to enrollment of potential participants at a given site, the study protocol was submitted together with its associated documents [e.g., Informed Consent Form (ICF), questionnaires, and communication materials] to the responsible IEC for its review. The written favorable opinion/approval of the IEC was provided to each study physician, and a copy was filed in the Study Master File. Pertinent safety information was submitted to the relevant IRB/IECs during the course of the study in accordance with local regulations and requirements.

## Author Contributions

AA, ES, and MS: analysis, interpretation, and drafting the manuscript. AA, AH, LvdM, BL, and AKH: conception and design. AH, LvdM, BL, and AKH: revising the manuscript. BL: research group supervision. AKH: data acquisition.

## Conflict of Interest Statement

The authors declare that the research was conducted in the absence of any commercial or financial relationships that could be construed as a potential conflict of interest.

## Footnotes

1 https://www.enroll-hd.org/participate/clinic-locations/

2 https://www.enroll-hd.org/ethics/

## Appendix

**HDQoL-C:** Please respond to each question – check the box that best reflects your opinions.

******		**10**	**9**	**8**	**7**	**6**	**5**	**4**	**3**	**2**	**1**
		
		**(Strongly agree)**									**(Strongly disagree)**
	**Section 1**										
1.	How satisfied are you with feeling a part of your social environment?										
2.	How satisfied are you with your relationships with your friends?										
3.	How satisfied are you with your psychological health?										
4.	How satisfied are you with what you have achieved in life?										
5.	How satisfied are you with family relationships?										
6.	How satisfied are you with your physical health?										
7.	How satisfied are you with the professional support you receive?										
8.	How satisfied are you with the medical treatment that your HD relative(s) receive(s)?										
9.	How satisfied are you with the way other people behave toward the HD person?										
	**Section 2**										
1r.	I feel sad										
2r.	I feel depressed**										
3r.	I feel a sense of grieving										
4r.	I feel a sense of loss										
5r.	I feel stressed										
6r.	I feel exhausted										
7r.	I feel a sense of anguish										
8r.	I feel full of fear										
9r.	I feel lonely										
10r.	I feel guilty										
11r.	I feel isolated										
12r.	I feel financially disadvantaged										
13.	I feel I get enough sleep										
14r.	I feel worried about the genetic consequences of HD										
15.	I feel that HD has made me a stronger person										
16.	I feel supported										
17.	I feel that my role as a carer is rewarding										
18.	I feel satisfied with my overall QoL										
19.	I feel that I can cope										
20.	I feel there is hope for the future										
21.	I feel comforted by my beliefs (religious, philosophical, or spiritual)										
22.	I feel I have somebody to turn to for assistance if I am overwhelmed										
23.	I feel happy										
24.	I feel that HD brought something positive to my life.										
25.	I feel safe.										
26.	I feel that my own needs are important to others.										
27.	I feel I have enough time for myself.										
28r.	I feel embarrassed by the behavior of my HD relative(s).										
29r.	I feel ashamed of the behavior of my HD relative(s).										
30r.	I feel restricted by having to provide continuous care.										
31r.	I feel restricted by the need to maintain secrecy about HD in the family.										
32r.	I feel resentful.										
33r.	I feel threatened.										
34r.	I feel restricted by a regimented daily routine.										
35r.	I feel that I have had a duty of care forced on me.										
36r.	I feel frustrated by the discrimination of others toward my HD relative(s).										
37r.	I feel frustrated by the misconceptions of others toward my HD relative(s).										
38r.	I feel like I don’t know who I am anymore.										

*Scoring Key. Section 1. Satisfaction with life. Factor 1. Sum Questions 1–6 and calculate the mean. Factor 2. Sum Questions 7–9 and calculate the mean. Section 2. Feelings about living with HD. Factor 1. Sum Questions 1–14 and calculate the mean. Factor 2. Sum Questions 15–27 and calculate the mean. Factor 3. Sum Questions 28–36 and calculate the mean. Items with an *r* are reverse coded such that a score of 10 = 1, 9 = 2, 8 = 3, …, 1 = 10.*

**HDQoL-Cs:** Please respond to each question – check the box that best reflects your opinions.

******		**10**	**9**	**8**	**7**	**6**	**5**	**4**	**3**	**2**	**1**
		
		**Strongly agree**									**Strongly disagree**
	**Section 1**										
1.	How satisfied are you with feeling a part of your social environment?										
2.	How satisfied are you with your relationships with your friends?										
3.	How satisfied are you with your psychological health?										
4.	How satisfied are you with what you have achieved in life?										
5.	How satisfied are you with family relationships?										
6.	How satisfied are you with your physical health?										
7.	How satisfied are you with the professional support you receive?										
8.	How satisfied are you with the medical treatment that your HD relative(s) receive(s)?										
	**Section 2**										
1r.	I feel sad										
2r.	I feel depressed**										
3r.	I feel a sense of grieving										
4r.	I feel a sense of loss										
5r.	I feel exhausted										
6r.	I feel stressed										
7r.	I feel a sense of anguish										
8r.	I feel full of fear										
9.	I feel that I can cope										
10.	I feel there is hope for the future										
11.	I feel satisfied with my overall QoL										
12.	I feel supported										
13.	I feel comforted by my beliefs (religious, philosophical, or spiritual)										
14.	I feel that my role as a carer is rewarding										
15.	I feel safe										

*Scoring Key. Section 1. Satisfaction with life. Factor 1. Sum Questions 1–8 and calculate the mean. Section 2. Feelings about living with HD. Factor 1. Sum Questions 1–8 and calculate the mean. Factor 2. Sum Questions 9–15 and calculate the mean. Items with an *r* are reverse coded such that a score of 10 = 1, 9 = 2, 8 = 3, …, 1 = 10.*
